# Familial Environment and Overweight/Obese Adolescents’ Physical Activity

**DOI:** 10.3390/ijerph16142558

**Published:** 2019-07-17

**Authors:** Nicole S. Carbert, Mariana Brussoni, Josie Geller, Louise C. Mâsse

**Affiliations:** 1 School of Population & Public Health, University of British Columbia, Vancouver, BC V6T 1Z3, Canada; 2 British Columbia Children’s Hospital Research Institute, Vancouver, BC V5Z 4H4, Canada; 3 Department of Pediatrics, University of British Columbia, Vancouver, BC V6H 3V4, Canada; 4 St Paul’s Eating Disorder Program, Vancouver, BC V6Z 1Y6, Canada; 5 Department of Psychiatry, University of British Columbia, Vancouver, BC V6T 2A1, Canada

**Keywords:** physical activity, family environment, adolescents, parenting practices, moderators

## Abstract

(1) Background: Family environments can impact obesity risk among adolescents. Little is known about the mechanisms by which parents can influence obesity-related adolescent health behaviours and specifically how parenting practices (e.g., rules or routines) and/or their own health behaviours relate to their adolescent’s behaviours. The primary aim of the study explored, in a sample of overweight/obese adolescents, how parenting practices and/or parental modeling of physical activity (PA) behaviours relate to adolescents’ PA while examining the moderating role of parenting styles and family functioning. (2) Methods: A total of 172 parent-adolescent dyads completed surveys about their PA and wore an accelerometer for eight days to objectively measure PA. Parents completed questionnaires about their family functioning, parenting practices, and styles (authoritative and permissive). Path analysis was used for the analyses. (3) Results: More healthful PA parenting practices and parental modeling of PA were both associated with higher levels of adolescents’ self-reported moderate-vigorous physical activity (MVPA). For accelerometer PA, more healthful PA parenting practices were associated with adolescents’ increased MVPA when parents used a more permissive parenting style. (4) Conclusions: This study suggests that parenting practices and parental modeling play a role in adolescent’s PA. The family’s emotional/relational context also warrants consideration since parenting style moderated these effects. This study emphasizes the importance of incorporating parenting styles into current familial interventions to improve their efficacy.

## 1. Introduction

Over the last three decades, a marked increase in the prevalence of overweight or obese Canadian adolescents has raised concerns [1,2]. To help manage this ongoing problem, research suggests that engaging in positive health behaviours such as increased physical activity (PA) among other behaviours can act as a protective factor against obesity [3,4]. However, adoption of these weight-related health behaviours can be impacted by a number of proximal influences, including the family environment [5]. Indeed, there is evidence that even among children at genetic risk for developing obesity, family/home environment moderates their likelihood of developing obesity in childhood [6]. Therefore, understanding the familial factors that can influence behaviour change among overweight or obese adolescents is essential in order to target these powerful influences. 

Parents, in particular, can influence their children directly through parenting practices (i.e., rules or routines) and their own health behaviours, such as modeling PA. Parenting practices are specific actions or strategies parents use to help socialize their children’s behaviours [7,8]. In the context of PA, parental support in the form of emotional (e.g., encouragement) [9,10] and logistical (transportation to parks or playgrounds) [11,12] support have been positively associated with adolescents’ PA. In contrast, results have generally been mixed when examining parent PA (modeling) on adolescents’ PA [13,14]. For instance, accelerometer studies have found positive associations between parent and child/adolescent moderate-vigorous physical activity (MVPA) [14,15], while self-report studies remain inconsistent [16]. Together, these studies highlight the role of parents in influencing their adolescents’ behaviours. However, the majority of this research has involved children and adolescents of normal weight. Evidence exploring the relationship between parenting practices and overweight or obese adolescents’ PA is lacking [17]. 

In recent years, family context has emerged as an important factor in the formation of adolescents’ PA behaviours. Two main contextual elements of interest include parenting style and family functioning. Parenting style is the emotional climate in which parents raise their child or the way parents interact with their child [18]. According to Baumrind [19,20], three parenting styles exist, including authoritative, authoritarian, and permissive: Authoritative parents exercise control in a supportive and understanding way, by encouraging verbal interaction. Authoritarian parents exercise high control in the form of demands and obedience, while discouraging verbal interaction. Permissive parents exercise minimal control by giving in to their child’s demands and provide little to no structure. On the other hand, family functioning acts as an all-encompassing dimension that focuses on how family subsystems interact with one another in terms of their cohesion and flexibility to impact the entirety of behaviors in the family unit. Although research in this area is generally sparse, recent models suggest that since parenting styles and family functioning are considered to be contextual elements, they may function at a higher level and act as moderators [21,22]. Specifically, parenting style and family functioning have the ability to act as moderators and impact child development (e.g., PA behaviours) indirectly by changing the effectiveness of parenting practices and modeling behaviours [18,23]. As a result, how children react to and perceive their parents’ wishes/demands may stem from the broader familial environment [7,24]. More specifically, parenting styles and family functioning can attribute a positive or negative undertone to the strategies employed by parents. For instance, parents who exhibit a more controlling parenting style by setting strict boundaries on children’s outdoor play may be viewed by their child as heavily controlling if the exchange between the parent and child is such that parents enforce rules which the child must obey—ultimately hindering outdoor play. Alternatively, rule setting has the potential to be regarded as warm if the parent-child dynamic involves an age-appropriate discussion on the reasoning behind the rules, openness to change rules, etc. [25]. Moreover, Kitzmann and colleagues [18], allude to the idea that parents’ attempts to engage their children in activities may be more successful when they already enjoy interacting and spending time together as a family (high family functioning). As a result, children may adopt more positive PA behaviours than those families who do not spend much time together (low family functioning). However, more research into these higher-level dimensions is needed to understand the extent to which context promotes PA behaviours among adolescents who are overweight or obese. 

**Figure 1:**
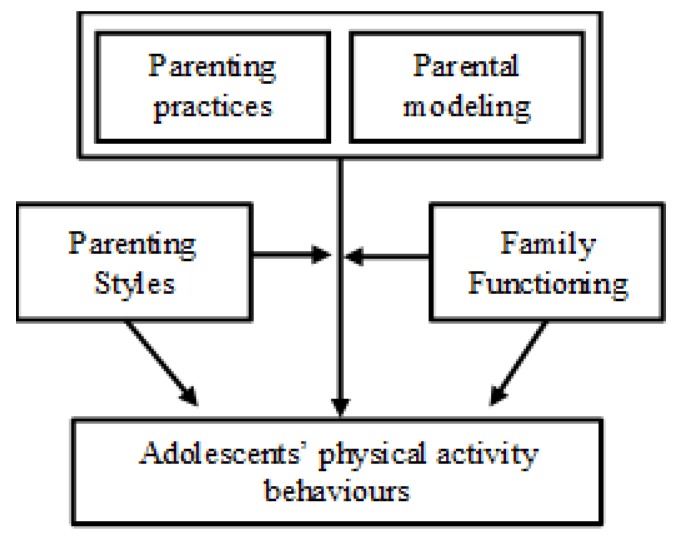
Theoretical model testing how parenting and family factors relate to adolescents’ physical activity behaviours.

Although prior studies have examined parenting practices and parental modeling independently with regards to adolescent PA, less is known whether both factors are jointly important or whether parenting styles and family functioning moderate these associations. Hence, the present study examines how parenting practices, parental modeling, and adolescent PA fit within these broader family-level components. The primary aim of the study (Figure 1) was to assess whether both parenting practices and parental modeling of PA are associated with adolescents’ PA, while examining the extent to which parenting styles and family functioning act as moderators. Given that PA parenting practices and parental modeling may be correlated, the secondary aims of this study assessed these relationships separately and examined: 1) The relationships between parenting practices adolescents’ PA behaviours while examining the role of parenting styles and family functioning as moderators, and 2) the relationships between parental modeling of PA and adolescents’ PA while examining the role of parenting styles and family functioning as moderators. Figure 1 presents conceptual relationships tested in this study guided by Bronfenbrenner’s ecological model [26] as well as suggestions from other frameworks [7,18,23,24,27], which considered the moderating role of parenting style and family functioning, under the assumption that the influence of specific PA parenting practices (e.g., logistic support, facilitation) and parental modeling (parent’s PA levels and self-report) on adolescent PA may be conditionally related to these higher level parental factors. 

## 2. Materials and Methods 

### 2.1. Study design

This is a secondary analysis of the baseline data collected as part of a study elucidating the individual and household factors that predict adherence to an e-health family-based lifestyle behaviours modification intervention for overweight/obese adolescents and their family [28].

### 2.2. Participants

Participants for the analyses included 172 parent/adolescent dyads who filled out a baseline measurement tool prior to starting an e-health family-based lifestyle behaviour modification intervention [28]. Among these families, 68% were recruited via advertisements (newspapers, parenting magazines, Facebook, Craigslist), 28% were previous patients of the British Columbia (BC) Children’s Hospital Endocrinology and Diabetes Clinic or Healthy Weight Shapedown program, and 5% were recruited via word of mouth. Parent-adolescent dyads were eligible to participate in the main study if the adolescent was overweight or obese according to the World Health Organization (WHO) cut-points [2] and the parent consented to take part in the study with them. Additional requirements included having internet at home, residing in the Greater Vancouver (BC) area, no plans to move during the study period (three years), and being literate in English. Adolescents were ineligible to participate in the study if they had any comorbidity (e.g., physical disability) that limited their ability to be physically active or eat a normal diet, a history of psychiatric problems or substance abuse, medication use that impacts body weight, or a Type 1 diabetes diagnosis.

### 2.3. Procedures

Ethics approval was obtained from the University of British Columbia and the University of Waterloo. At baseline, parents completed a number of online surveys that asked about their parenting practices, parenting styles, and family functioning. Adolescents and parents filled out a series of surveys on their PA habits. Additionally, adolescents and parents were required to wear an accelerometer (over their hip under their clothes) for eight full days following the baseline visit, during waking hours. Finally, adolescent-parent pairs were asked to keep track of their sleep duration and times when they were not wearing the accelerometer in the logbook. 

### 2.4. Measures

A series of self-report measures were used to capture parenting practices, parenting styles, and family functioning. Adolescent and parent PA were assessed using both self-report and objective measures. 

**Parenting practices (parent self-report):** A family nutrition and PA screening measure [29] was used to assess PA parenting practices. An exploratory factor analysis supported the one–factor structure of the original 15-item scale with a score that had adequate internal consistency (0.70) and was related to body mass index (BMI) categories of children [29]. A four-factor structure, composed of PA, eating, breakfast, and screen time was also supported (Cronbach’s alpha coefficients of 0.60, 0.64, 0.55, 0.33, respectively) to examine practices related to specific behaviours. Items consisted of two opposing statements in which parents selected the statement that applied to their child and/or family. For PA practices, three items asked whether the child participates in organized sports, whether the child is spontaneously active, and whether the family is active together. This response style was selected to normalize both positive and negative response options to minimize social desirability bias [30]. Responses were converted to a four-point numerical scale, and reverse coded as needed, so that a score of four indicated more healthful parenting practices. 

**Parenting styles (parent self-report):** Parenting styles were measured using a modified version of Cullen’s 16-item authoritative parenting scale [31]. The original measure includes two subscales measured on a four-point Likert scale ranging from never to always., namely authoritative (11 items) and negative (five items) parenting styles and has been previously tested in a sample of ethnically diverse parents and grade four to six students [31]. With regards to item variance, a principal component analysis (PCA) revealed that the authoritative subscale explained 30% while the authoritative subscale explained 11%. Cronbach’s alphas for the authoritative and negative subscales were 0.72 and 0.73, and yielded Pearson test-retest correlation coefficients of 0.53 and 0.82, respectively. However, as the structure in the study sample was not supported according to the initial confirmatory factor analysis (*X^2^* (*df* = 89) = 187.6, *p* < 0.00, RMSEA = 0.084 and 90% CI = 0.067–0.101, CFI = 0.844, SRMR = 0.080), the authoritative (e.g., “tell child he/she does a good job”, “tell child I like my child just the way he/she is”) and negative (e.g., “forget the rules I make for my child”, “hard to say no to child”) subscales were reduced from ten to three items respectively, along with the addition of two correlated error terms according to modification indices and conceptual relevance. As the content of the remaining three items on the negative parenting scale were more permissive in nature, the scale is referred to as measuring “permissive” parenting. In the present sample, confirmatory factor analysis supported the revised structure (*X^2^* (*df* = 62) = 109.8, *p* < 0.00, RMSEA = 0.070 and 90% CI 0.048–0.091, CFI = 0.919, SRMR = 0.067), and had a Cronbach’s alpha of 0.85 for authoritative and 0.59 for permissive. To derive indices, items were summed and dichotomized at the median to split parents into high/low authoritative and permissive style. A fairly even split was met for the authoritative style (72 participants allocated to the high group and 82 to the low group), but not for the permissive style (50 participants allocated to the high group and 120 to the low group) due to a majority of parents scoring at the median. 

**Family functioning (parent self-report):** The Family Adaptability and Cohesion Evaluation Scale IV (FACES IV) [32] assessed family functioning. The original measure comprises 42 items assessed on a five-point Likert scale ranging from strongly agree to strongly disagree. The measure contains six subscale: balanced cohesion (e.g., “feeling very close”), balanced flexibility (e.g., “able to adjust to change”), enmeshed (e.g., “spending too much time together”), disengaged (e.g., “avoid contact with each other”), chaotic (e.g., “never seem to get organized”), and rigid (e.g., “rules for every possible occasions”). These six subscales measured two overarching dimensions of cohesion and flexibility [32]. The six-factor structure was supported in a sample of US post-secondary adults (mean age: 28) and all scales had high internal consistency (Cronbach’s alpha 0.77 to 0.89) [32]. 

Using the conversion chart developed by Olson [32], raw scores for each family functioning subscale were transformed into subscale-specific percentile scores. Cohesion and flexibility ratio scores were computed independently based on percentile scores. Refer to Table 1 for the formulas used to compute the ratio scores. For analytic purposes, the cohesion ratio and flexibility ratio were dichotomized. Participants were classified into the high family functioning group if their ratio scores were above the median on both ratio scores. Those with scores below the median on at least one of these two Ratios were classified as low family functioning. Hence, those families which scored below 1.9 on the cohesion ratio and below 1.4 on the flexibility ratio were categorized as belonging to the high family functioning group.

**Accelerometer to measure MVPA (worn by child and parent):** Two types of accelerometers (Actigraph GT3X or GT3X+) were used to measure MVPA. Parental modeling (PA) was computed using parent MVPA as described below. Data from the Actigraph accelerometers was processed using a program in Stata that processed the data following previous recommendations [33,34]. Data from the accelerometers were collected in spans of 10 seconds and aggregated into one-minute intervals for the analyses. A day of recording was considered valid if the accelerometer was worn at least 10 hours per day, which represents 63% of the time participants are awake (for those who sleep eight hours). Non-wear time was described as a period of at least 60 minutes that resulted in no activity [33]. If participants had three valid days (including one weekend day) of wear time, they were included in the analyses. To help determine the appropriate minutes of MVPA, child and parent-specific MVPA cutoffs were used (≥2296 and ≥1952 accelerometer counts in a one-minute time frame, respectively) [35]. Counts above this cut point were combined to calculate total minutes of MVPA during the assessment week [36]. To determine the average minutes of MVPA at baseline, total MVPA was divided by number of days.

**Seven-day physical activity recall (PAR) to measure MVPA (interview-administered to child and parent separately):** The seven-day PAR is a semi-structured interview aimed at estimating the amount of MVPA the parent or child has engaged in for 10 minutes or longer in the seven days leading up to the interview. PA Parental modeling was also computed using parent self-report of MVPA as described below. The measure, which is adapted from the Stanford Five-City Project [37], is primarily used to record the intensity and duration minutes) of participants’ activities. To aid participants in identifying which level of intensity corresponded to the activity they performed, they were provided an overview of three different levels of intensity. These levels included leisure walking (i.e., relaxing walk), moderate activities (i.e., brisk walking) and very hard activities (i.e., running hard). In addition to the regular interview questions, probing methods were employed to ensure that sufficient information was obtained from each participant. The Compendium of Energy Expenditure for Youth [38] was used to assign the appropriate number of metabolic equivalents (where 1 MET is the amount of energy expended at resting) to each activity the participant performed. Self-reported MVPA time was defined as the average minutes per day spent performing activities that were ≥4 MET [35,39,40]. Time spent in MVPA was computed by summing all the activities above this point. The total minutes in a week was divided by seven to obtain average minutes of MVPA per day. 

### 2.5. Data Analysis

Path analysis was used to conduct all analyses in Stata 13. Full information maximum likelihood was employed to handle missing data. For all the analyses, two models were run: One model using adolescents’ MVPA measured with accelerometry as the dependent variable and another model using adolescents’ MVPA measured with self-report as the dependent variable. The analyses for the primary and secondary aims followed the same process: (1) Model 1 tested whether PA-related parenting practices and/or parental modeling (PA) were associated with adolescents’ MVPA, and (2) the final model included the relationships tested in Model 1 but added all the moderating variables (i.e., authoritative and permissive parenting styles as well as family functioning) and the relevant interaction terms as depicted in Figure 1. Note that interaction terms were then entered into the analysis one by one for each of the corresponding models and were kept in the model if p < 0.10. All variables were standardized prior to inclusion in models so as to address issues of convergence. Each model was adjusted for the following covariates: adolescent sex, adolescent age, and parental income. The secondary analyses are presented first as they serve to interpret and build the model for the main aim of this study.

To examine assumptions of linear regression, residual plots and bivariate scatterplots were estimated for each model. The magnitude, indicated by the standard coefficient (SC) of a path, as well as the associated p-value, were examined to determine the significance of the path.

## 3. Results

The analytic sample is characterized in Table 2. As shown in Table 3, adolescents and parents accumulated around half an hour of MVPA per day as measured by accelerometry and about 56 and 69 minutes of self-reported MVPA per day, respectively. The majority of parents had high scores on the authoritative parenting style scale and midrange on the permissive parenting scales, as most scored 6.0 on a scale ranging from 3 to 12. Regarding family functioning, most parents were balanced on the cohesion and flexibility ratios as the mean ratios both exceeded one. 

**Figure 2:**
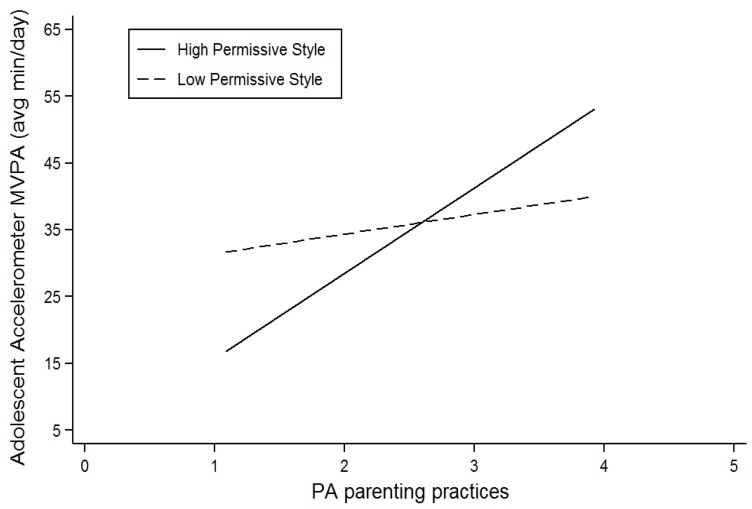
Graph illustrating how associations between adolescents’ amount of moderate-vigorous physical activity (MVPA) and physical activity (PA) parenting practices were moderated by a permissive parenting style.

Table 4 displays associations between PA parenting practices and adolescents’ MVPA and whether associations were moderated by parenting styles and family functioning. As demonstrated in Table 4, Model 1 (without moderators) highlights that PA parenting practices were significantly associated with adolescents’ self-report of MVPA and that there was a trend towards significance (*p* = 0.06) with adolescents’ MVPA measured by accelerometry. Specifically, more healthful PA parenting practices were associated with higher levels of adolescents’ MVPA. When the moderators were included in the model, the interaction term between permissive style and PA parenting practices became significant. In contrast, PA parenting practices was the only significant predictor for adolescents’ self-report MVPA when the moderators were added. Figure 2 illustrates the interaction of permissive style by PA parenting practices, suggesting that more healthful PA parenting practices were positively associated with adolescents’ MVPA but also indicating that this association was more pronounced among adolescents whose parents use a high permissive style compared to those with a low permissive style. As shown in the graph, however, the direction of this association reverses when parents employ less healthful PA practices. 

In all models, adolescents’ sex was the only significant covariate. The results suggest that adolescent boys had significantly higher MVPA than adolescent girls and this was observed for both accelerometry and self-report assessment of MVPA (Table 3).

Table 5. displays associations between parental modeling of PA and adolescents’ MVPA, as well as whether parenting styles and family functioning moderated these associations. As shown in Table 5, Model 1 (without moderators) highlights that parental modeling of PA was significantly associated with adolescents’ MVPA for both accelerometer and self-report. Specifically, parents who modeled high levels of PA were associated with increased PA among overweight/obese adolescents. When the moderators were added into these models, no significant effects emerged for parenting styles or family functioning, but parental modeling of PA remained significant.

Table 6 displays the association of both PA parenting practices and parental modeling of PA on adolescents’ MVPA and whether parenting styles and family functioning moderate these associations. Model 1 (without moderators) highlights that PA parenting practices and parental modeling of PA were significantly associated with self-report of MVPA. Specifically, more healthful PA parenting practices and parental modeling of PA were both associated with higher levels of adolescents’ MVPA. Although parental modeling of PA (accelerometry) was significantly associated with adolescents’ MVPA measured with accelerometry in Model 1, a trend towards significance (p = 0.07) was observed for this relationship in the final model. When the moderators were added into the model, a significant interaction between permissive style and PA practices was observed. However, this was only observed for MVPA measured with accelerometry. This is similar to our findings reported in Table 4. This finding is illustrated in Figure 2 (see previous figure reported and description) 

## 4. Discussion

The purpose of this study was to examine the effect of parenting practices and/or parental modeling on the PA behaviours of overweight/obese adolescents and explore whether parenting styles and family functioning act as moderators. With regards to the primary aim of the study, when considering both PA parenting practices (i.e., facilitation, logistic support) and parental modeling of PA (i.e., PA self-report), both were significantly associated with adolescents’ self-report of MVPA—where higher MVPA occurred in families that had more positive parenting practices and modeled an active lifestyle In addition, a significant interaction between permissive style and PA parenting practices emerged for adolescents’ MVPA measured with accelerometry—where permissiveness was found to amplify the association between healthy PA parenting practices and adolescents’ MVPA. Interestingly, family functioning did not emerge as an important moderator. The findings were similar when PA parenting practices and parental modeling were examined independently (secondary aims), except that the association between parental modeling of PA and adolescents’ MVPA measured with accelerometry was significant instead of being borderline significant. Overall, the results highlight the importance of healthy PA parental practices and modeling to support overweight/obese adolescents’ MVPA as well as the role of permissiveness in further supporting their engagement in PA.

Given that most of the literature has focused on the influence of parenting practices and modeling separately [12,15,41,42], the present study revealed that parenting practices and parental modeling together, may be important factors in overweight/obese adolescent PA behaviours. The few studies that have explored both practices and modeling together in the context of PA report conflicting results in comparison to the present study. Previous studies have reported that the importance of modeling is diminished by other constructs, such as parental encouragement and support [43,44,45]. For instance, a study conducted among grade 7–12 students found parenting practices, namely parental support, to be more influential than parental modeling [43]. However, these studies targeted a general sample of adolescents while the present study focused on overweight/obese adolescents, which may explain the discrepancies. It may be that parents who are more active or model an active lifestyle are in a better position to support their overweight/obese adolescents’ PA as they can, for example, be active together. On the other hand, adolescents who are not overweight/obese may only need support from their parents to be physically active, such as transportation to a playground, while overweight/obese adolescents may need the additional modeling component to enhance their drive and motivation to be active. Therefore, the combination of parental modeling along with specific parenting practices such as taking the child to an appropriate location for PA or encouragement may be necessary to influence the activity of overweight/obese adolescents. 

Evidence to support the hypothesis that family functioning would act as a moderator on the relationship between parenting practices and/or parental modeling and adolescents’ physical activity, was not found. Of note, few studies to date have explored the role of family functioning as a moderator [18,46]. In a sample of healthy adolescents, one study found evidence of family functioning as a moderator on the relationship between family meals and unhealthful weight management behaviours [46]. Despite no literature exploring family functioning as a moderator within the context of child obesity, a review has provided some indirect evidence to help support this notion [18]. As pointed out by correlational findings, overweight/obese children have a greater likelihood of experiencing more family conflict and less family cohesion compared to their normal-weight counterparts [47,48]. Although the directionality of this effect remains unclear, these correlations suggest that in families where an overweight child is present, more support may be needed to help establish or manage positive health behaviours [18]. Although this review provides some reasoning to support the moderating effect of family functioning on adolescent health behaviours, the evidence base remains unclear [18,24]. In the present study, it is important to note that the null findings apparent for family functioning may be a result of the sample’s characteristics. Of note, families in our sample were predominantly balanced in cohesion and flexibility. Therefore, families categorized as high or low functioning may be quite similar to one another. Thus, future research should strive to capture families that truly fit into the high or low family functioning groups to better understand the true potential of family functioning.

The association between parenting practices on adolescents’ PA behaviours was moderated by parenting styles, however, it was only partially consistent with the study hypotheses. This finding highlights that the moderating effect of parenting styles on the association between parenting practices on adolescents’ PA behaviours was more complex than anticipated. Two other studies have reported similar results, suggesting that more healthful practices performed in a more permissive way are associated with more adolescent MVPA [25,49]. According to Hennessy and colleagues, two types of PA parenting practices (monitoring and reinforcement) were associated with child accelerometer PA when expressed in the context of a permissive parenting style [25]. Similar findings were also observed by Langer and colleagues who found parental support was only associated with adolescent PA when expressed in the context of a permissive parenting style [49]. One potential explanation for this finding may be that permissive parenting characterized by high warmth and low demand is associated with more unstructured playtime and more enjoyable activities [50]. Therefore, being permissive in the context of PA may provide adolescents with more free time for active play and if they feel encouraged and supported by their parent with respect to PA, they may choose to be physically active.

The association between PA parenting practices, styles, and adolescents’ MVPA was only observed when adolescents’ MVPA was measured by accelerometry. While both accelerometer and self-report measures have been validated to assess PA, there are clear differences in the two measures. For instance, accelerometer data give more accurate estimates of walking-based activities and avoid many of the issues that go along with self-report, such as recall and response bias [51]. However, it is important to highlight that accelerometers are unable to capture certain types of activities, such as swimming and activities involving the use of upper extremities. Compared to direct measures, self-report methods appear to estimate greater amounts of higher intensity (i.e., vigorous) PA than in the low-to-moderate levels [51]. The main difference in the present study is that the self-report MVPA interaction with parenting practices and styles did not appear while it was found with the accelerometer. Measurement error with self-report tends to be higher, as noted by the increased chance of recall and response bias, which may lead to decreased power and perhaps explain why a significant interaction was not observed with the self-report data.

The study has some limitations that should be considered. First, it is difficult to assume a cause and effect relationship due to the cross-sectional nature of the study. For instance, relationships observed in this study may be bi-directional since both parents and children can shape one another [52,53]. Second, measurement errors may have biased study results. MVPA was assessed with both subjective (self-report) and objective (accelerometer) measures. Self-report measures are subject to reporting biases, such as recall and social desirability bias, since individuals are known to have poor recall of past PA levels and tend to overestimate their PA (biased reporting and low validity), respectively [54,55,56]. Therefore, inconsistency in our results may be due to these various forms of measurement error. Third, the parenting measures had some measurement challenges. Of the three parenting styles developed by Baumrind, our study only captured two (authoritative and permissive). Therefore, this may have limited our ability to adequately capture the parenting style of each parent. Additionally, as all parenting measures (parenting practices, parenting styles, and family functioning) were self-reported, our results may have been impacted by social desirability bias. As such, this may have led to an overestimation of the results. Given the strong evidence of familial clustering of obesity [57,58], our study may have benefited from information on parental BMI. Including this variable in our analyses may have attenuated our results, given its association with the family PA environment as well as various parenting behaviours (e.g., monitoring child PA, setting limits for PA) [59]. Finally, our sample included adolescent volunteers who were classified as overweight or obese and were willing to take part in an e-health intervention. Despite the fact that our results may not be generalizable to the general population, it is important to consider overweight/obese adolescents not only because they are typically understudied, but because they are frequently targeted by treatment interventions. 

This is the first study to explore the moderating effects of both parenting styles and family functioning on adolescents’ PA behaviours. It is one of the only studies to examine moderating effects in a sample of overweight or obese adolescents, which is essential when trying to design effective weight-management interventions. Understanding how parenting practices and modeling interact with styles and functioning on adolescents’ health behaviours provides useful information for the development of familial interventions. It is also one of the few to use both accelerometers and self-report to directly measure and compare both parent and adolescents’ PA levels.

Findings from this study offer implications for intervention development. First, interventionists (e.g., nurse practitioners) should consider parenting factors when counselling families with an overweight or obese adolescent. As part of family-based interventions, interventionists should encourage parents to not only provide support for their child’s PA, but modify their own PA. Secondly, family context, specifically, parenting style, may help improve the efficacy of family-based interventions. For example, interventionists could teach parents that parenting styles and practices go hand in hand and elicit different PA behaviours from their adolescents.

## 5. Conclusions

In conclusion, the study emphasizes the need to consider both parenting practices and parental modeling in shaping overweight/obese adolescents’ PA behaviours, as well as acknowledges the importance of using such parenting tactics in the appropriate context. 

## Figures and Tables

**Table 1:** Formulas for ratio scores.

Ratios	Scores (Use Percentiles)
Cohesion Ratio	Balanced Cohesion/ ((Enmeshed + Disengaged)/2)
Flexibility Ratio	Balanced Flexibility/ ((Chaotic + Rigid)/2)

**Table 2:** Demographic characteristics of adolescents and their parents.

	%	Mean ± SD	Range
Adolescent age, in years (n = 172)		13.1 ± 1.8	11.0–16.0
Adolescent sex, female (n = 172)	55.2		
Adolescent BMI z-score (n = 172)		2.70 ± 0.83	1.1–6.0
Parent age, year (n = 172)		45.7 ± 6.2	31–66
Parent sex, female (n = 172)	84.3		
Parent BMI (n = 172)		30.3 ± 7.3	18.3–69.0
**Household income in CAD** (n = 169)			
$60,000 or less	34.9	--	--
$60, 000– $100,000	33.7	--	--
$100, 001 or more	31.4	--	--
**Parent education** (n = 172)			
High school or less	17.4	--	--
Trade certificate/diploma	41.3	--	--
Bachelor degree	18.6	--	--
More than bachelor degree	22.7	--	--
**Parental marital status** (n = 172)			
Married or Common-law	70.9	--	--
Single/Widowed/Separated/Divorced	29.1	--	--
**Ethnicity** (n = 171)			
White	48.0	--	--
East or Southeast Asian	13.5	--	--
South Asian	12.3	--	--
Aboriginal	10.0	--	--
Other ethnicity	16.4	--	--

BMI = Body mass index, SD = Standard deviation, % = Percentage, CAD = Canadian dollars.

**Table 3:** Description of variables in models.

	n	Mean ± SD	Range
**Independent Variables**			
*Parenting Practices*			
Physical activity	160	2.4 ± 0.8	1–4
*Parental Modeling*			
Measured MVPA (min/day)	155	29.3 ± 20.3	1.0–103.1
Self-reported MVPA (min/day)	170	68.9 ± 89.7	0.0–488.6
**Dependent Variables**			
*Adolescent Health Behaviours*			
Measured MVPA (min/day)	131	34.4 ± 20.9	2.8–119
Self-reported MVPA (min/day)	158	56.1 ± 47.7	0.0–272.1
**Moderators**			
*Parenting Style*			
Authoritative	160	34.8 ± 4.3	21–40
Permissive	170	6.0 ± 1.4	3–11
*Family Functioning*			
Cohesion ratio	168	2.1 ± 0.63	0.88–4.4
Flexibility ratio	162	1.4 ± 0.35	0.68–3.1

MVPA = moderate-vigorous physical activity.

**Table 4:** Association between physical activity (parenting practices of physical activity) (PA) and adolescents’ accelerometer and self-report of moderate-vigorous physical activity (MVPA).

Accelerometer MVPA Time				Self-Reported MVPA Time	
		Model 1	Final Model	Model 1	Final Model
		SC (SE)	SC (SE)	SC (SE)	SC (SE)
Independent Variable	PA Practices	0.17 (0.09) *p* = 0.06	0.09 (0.11) *p* = 0.43	0.38 (0.08) *p* = 0.00	0.38 (0.08) *p* = 0.00
Moderators	Authoritative Style	--	−0.15 (0.09) *p* = 0.10	--	0.01 (0.08) *p* = 0.85
	Permissive Style	--	−0.07(0.09) *p* = 0.46	--	−0.06 (0.08) *p* = 0.41
	Family Functioning	--	−0.05 (0.09) *p* = 0.53	--	0.12 (0.08) *p* = 0.15
	Authoritative Style* PA Practices	--	R	--	R
	Permissive Style* PA Practices	--	0.23 (0.11) *p* = 0.03	--	R
	Family Functioning* PA Practices	--	R	--	R
Covariates	Adolescent sex	0.22 (0.08) *p* = 0.01	0.21 (0.08) *p* = 0.01	0.17 (0.07) *p* = 0.02	0.18 (0.07) *p* = 0.02
	Adolescent age	0.00 (0.09) *p* = 0.98	−0.06 (0.09) *p* = 0.53	0.02 (0.08) *p* = 0.76	0.06 (0.08) *p* = 0.47
	Parent income	−0.06 (0.09) *p* = 0.46	−0.02 (0.09) *p* = 0.83	0.01 (0.08) *p* = 0.88	−0.03 (0.08) *p* = 0.67

Footnote: SC = Standardized coefficient, SE = Standard error, R = Removed interaction terms from model if *p* > 0.10 to ensure parsimony. Model 1: Examine relationship between PA parenting practices and adolescents’ MVPA while controlling for adolescent sex, adolescent age, parental income Final Model: Adds to Model 1 test of moderation and removes interaction terms *p* > 0.1. * Refers to interaction term.

**Table 5:** Association between parental modeling of physical activity (PA) (accelerometer and self-report) and adolescents’ accelerometer and self-report of moderate-vigorous physical activity (MVPA).

Accelerometer MVPA				Self-report MVPA	
		Model 1	Final	Model 1	Final
		SC(SE)	SC(SE)	SC(SE)	SC(SE)
Independent Variable	Modeling (PA)	0.22 (0.08) *p* = 0.00	0.21 (0.08) *p* = 0.01	0.29 (0.08) *p* = 0.00	0.30 (0.07) *p* = 0.00
Moderators	Authoritative Style	--	−0.06 (0.09) *p* = 0.54	--	0.09 (0.08) *p* = 0.27
	Permissive Style	--	−0.02 (0.09) *p* = 0.83	--	−0.07 (0.08) *p* = 0.38
	Family Functioning	--	0.00 (0.10) *p* = 0.99	--	0.12 (0.09) *p* = 0.18
	Authoritative Style* Parental Modeling PA	--	R	--	R
	Permissive Style* Parental Modeling PA	--	R	--	R
	Family Functioning* Parental Modeling PA	--	R	--	R
Covariates	Adolescent sex	0.19 (0.08) *p* = 0.02	0.23 (0.08) *p* = 0.00	0.23 (0.07) *p* = 0.00	0.23 (0.07) *p* = 0.00
	Adolescent age	−0.04 (0.09) *p* = 0.63	−0.05 (0.09) *p* = 0.57	−0.07 (0.08) *p* = 0.35	−0.02 (0.08) *p* = 0.78
	Parent income	−0.05 (0.09) *p* = 0.60	−0.06 (0.09) *p* = 0.54	0.08 (0.08) *p* = 0.33	0.02 (0.08) *p* = 0.80

SC = Standardized coefficient, SE = Standard error, R = Removed interaction terms from model if *p* > 0.10 to ensure parsimony. Model 1: Relationship between parental modeling (PA) and adolescents’ amount of MVPA while controlling for adolescent sex, adolescent age, parental income. Final Model: Adds to Model 1 test of moderation and removes interaction terms *p* > 0.1. *Refers to interaction term.

**Table 6:** Associations between parenting practices and parental modeling of physical activity (PA) on adolescents’ accelerometer and self-report of moderate-vigorous PA (MVPA).

Accelerometer PA				Self-Report PA	
		Model 1	Final Model	Model 1	Final Model
		SC (SE)	SC (SE)	SC (SE)	SC (SE)
**Independent** **Variables**	PA Practices	0.12 (0.09) *p* = 0.19	0.04 (0.11) *p* = 0.75	0.33 (0.08) *p* = 0.00	0.33 (0.08) *p* = 0.00
	Modeling (PA)	0.19 (0.08) *p* = 0.02	0.16 (0.09) *p* = 0.07	0.22 (0.08) *p* = 0.00	0.23 (0.07) *p* = 0.00
**Moderators**	Authoritative Style	--	−0.12 (0.09) *p* = 0.20	--	0.03 (0.08) *p* = 0.71
	Permissive Style	--	−0.04 (0.09) *p* = 0.64	--	−0.09 (0.08) *p* = 0.22
	Family Functioning	--	−0.01 (0.10) *p* = 0.91	--	0.12 (0.08) *p* = 0.15
	Authoritative Style* PA Practices	--	R	--	R
	Permissive Style* PA Practices	--	0.23 (0.11) *p* = 0.03	--	R
	Family Functioning* PA Practices	--	R	--	R
	Authoritative Style* Modeling (PA)	--	R	--	R
	Permissive Style* Modeling (PA)	--	R	--	R
	Family Functioning* Modeling (PA)	--	R	--	R
**Covariates**	Adolescent sex	0.22 (0.01) *p* = 0.01	0.21(0.08) *p* = 0.00	0.18 (0.07) *p* = 0.01	0.19 (0.07) *p* = 0.00
	Adolescent age	−0.01 (0.09) *p* = 0.89	−0.05 (0.09) *p* = 0.53	0.00 (0.08) *p* = 0.90	0.05 (0.08) *p* = 0.53
	Parent income	−0.06 (0.09) *p* = 0.48	−0.03 (0.09) *p* = 0.75	0.05 (0.07) *p* = 0.55	−0.01 (0.08) *p* = 0.92

SC = Standardized coefficient, SE = Standard error, R = Removed interaction terms from model if *p* > 0.10 to ensure parsimony. Model 1: Relationship between PA parenting practices and parental modeling of PA via accelerometer/self-report on adolescents’ amount of physical activity (accelerometer/self-report) while controlling for adolescent age, adolescent sex, and parental income. Final Model: adds to Model 1 test of moderation and removes interaction terms *p* > 0.1. * refers to interaction term.
